# Translation, adaptation and validation of an epilepsy screening instrument in two Ghanaian languages

**DOI:** 10.1371/journal.pone.0303735

**Published:** 2025-01-17

**Authors:** Emmanuel Kwame Darkwa, Sabina Asiamah, Elizabeth Awini, Cynthia Sottie, Anthony Godi, John E. Williams, Albert Akpalu, J. Helen Cross, Josemir W. Sander, Arjune Sen, Charles R. Newton, Anthony Danso-Appiah, Patrick Adjei

**Affiliations:** 1 Dodowa Health Research Centre, Research and Development Division, Accra, Ghana; 2 Ghana Health Service, Accra, Ghana; 3 Department of Epidemiology and Disease Control, School of Public Health, University of Ghana, Legon, Accra; 4 University of Ghana Medical School, Accra, Ghana; 5 Korle Bu Teaching Hospital, Korle Bu, Accra, Ghana; 6 UCL Great Ormond Street NIHR BRC Institute of Child Health, London, United Kingdom; 7 UCL Queen Square Institute of Neurology, London WC1N 3BG & Chalfont Centre for Epilepsy, Chalfont St Peter, United Kingdom; 8 Stichting Epilepsie Instellingen Nederland (SEIN), Heemstede, The Netherlands; 9 Department of Neurology, West China Hospital, Sichuan University, Chengdu, China; 10 Nuffield Department of Clinical Neurosciences, Oxford Epilepsy Research Group, John Radcliffe Hospital, Oxford, United Kingdom; 11 Department of Psychiatry, University of Oxford, Oxford, United Kingdom; 12 Centre for Evidence Synthesis and Policy, University of Ghana, Legon, Accra; University of Catania Department of Surgical and Medical Sciences Advanced Technologies GF Ingrassia: Universita degli Studi di Catania Dipartimento di Scienze Mediche Chirurgiche e Tecnologie Avanzate GF Ingrassia, ITALY

## Abstract

**Introduction:**

The prevalence of epilepsy in sub-Saharan Africa varies considerably, and the exact estimate for Ghana remains unclear, particularly in peri-urban areas where data are scarce. More community-based studies are required to understand better the actual burden of epilepsy in these areas and the difficulties in accessing healthcare.

**Objective:**

To adapt and validate a household survey epilepsy-screening instrument in Shai-Osudoku and Ningo-Prampram District of Greater Accra Region, Ghana.

**Methods:**

We developed a 17-item epilepsy screening instrument by modifying previously validated English language questionnaires. We included questions that could identify convulsive and non-convulsive seizures. Language experts forward- and back-translated the questionnaires into the two languages: Asante Twi and Dangme. Cases were people with confirmed epilepsy attending healthcare facilities where these languages are used. Controls were unaffected relatives of cases or people attending the same healthcare facilities for other medical conditions. We matched cases and controls for geographical location and ethnicity. An affirmative response to one of the seventeen questions by a participant was deemed a positive screen. The questionnaires were divided into two stages. The first stage consisted of broader, more general questions aimed at identifying potential cases of epilepsy. The second stage involved a more detailed and focused set of questions administered to those who screened positive in the first stage.

**Results:**

One hundred and forty Dangme speakers (70 cases and 70 controls) and 100 Asante Twi speakers (50 cases and 50 controls) were recruited. The sensitivity and specificity for Dangme were: Stage 1, 100% and 80%, and Stage 2, 98.6% and 85.7%. The Dangme version reliably identified epilepsy with positive predictive values of 83.3% and 87.3% at stages 1 and 2. The questionnaire excluded epilepsy with 100% and 98.4% negative predictive values. For the Asante Twi version, the sensitivity and specificity were 98% and 92% (95% at Stage 1, and for Stage 2, 96% and 94%. The Asante Twi questionnaire reliably specified epilepsy with positive predictive values of 92.5% and 94.1% at stages 1 and 2. It excluded epilepsy with negative predictive values of 97.9% and 95.9% for the two stages

**Conclusions:**

Our questionnaire is valid for the two languages and usable for community-based epilepsy surveys in Ghana. It can also be adapted for other resource-poor settings, although translation and iterative in-country testing will be needed to ensure its validity.

## 1. Introduction

Epilepsy is one of the most prevalent neurological diseases, affecting over 50 million people of all ages globally [1,2]. Estimates suggest that about 80% of people with epilepsy live in a resource-poor setting with a treatment gap of about 80% [3]. The burden of the disease is disproportionately weighted towards Africa, where more than 10 million people live with epilepsy [4,5]. In Ghana, the exact prevalence of epilepsy is unknown, but a previous estimation in a rural area suggested it to be 10 per 1000 or 1% of the population [6]. There are no data on burden in urban and peri-urban areas in Ghana.

Africa suffers from a chronic shortage of human resources for health more than any other region [7], with about 0.043 neurologists per 100,000 inhabitants [8]. This lack of personnel has led to task shifting, where skilled staff support and supervise less experienced healthcare workers to provide care at the primary healthcare level [9]. Screening questionnaires are needed to identify people with epilepsy in primary healthcare facilities.

Several epidemiologic surveys of epilepsy have been performed in Low and Middle-income Countries (LMICs) to estimate the prevalence [10]. These surveys often adopt a two-stage study design consisting of a screening phase during which trained field staff interview the population under investigation through a face-to-face meeting with participants. In the subsequent second phase, specialists clinically evaluate positive subjects to confirm true positives [11,12]. The choice of screening questions is essential, and to validate an instrument is mandatory to establish a screening test’s potential accuracy or inaccuracy. Questions targeting symptoms are vital to maximising sensitivity (i.e., the proportion of actual cases in the population that test or screen positive), especially in a resource-poor setting with limited access to healthcare. This is important as systematically acquiring clinical information significantly increases the performance of the questionnaire, especially when electroencephalography is unavailable [12].

In epilepsy care and management, antiseizure medication and further interventions depend on the type of seizure, epilepsy syndrome, comorbidity, and other considerations [13]. Solving the problem of epilepsy in resource-poor settings starts with identifying people with the disease. To this end, developing reliable and accessible screening tools has gained attention [14]. Primary or non-specialist healthcare workers may use an effective screening tool to identify individuals requiring intervention. These screening questionnaires should be in the respondents’ native language and culturally appropriate.

Translating an instrument to the target language must follow pre-determined guidelines [15]. Establishing a tool’s sensitivity, specificity, and predictive value is essential and emphasises the need for earlier validation [16]. Healthcare providers at the primary and secondary levels of care face many challenges without clinical algorithms or pragmatic rapid diagnostic tests [17]. There may be the need to enforce the widespread use of epilepsy screening questionnaires in primary and secondary healthcare settings to obtain critical information for clinical management and care as a witness. Information from relatives may be inaccurate [18].

We developed an epilepsy screening tool for household surveys. We sought to determine if the tool would improve the identification of all forms of epilepsy in selected health facilities in the Greater Accra Region of Ghana

## 2. Methods

### 2.1 Study population

Our population included children and adults consecutively enrolled from the Shai-Osudoku District Hospital, Prampram Polyclinic, Ningo Health Centre, and the Korle-Bu Teaching Hospital. The cases were individuals with confirmed diagnoses of epilepsy by a neurologist or physician with epilepsy expertise. Active epilepsy was operationally defined as “two or more unprovoked epileptic seizures separated by at least 24 hours within the last year” [19]. Controls were people attending the same hospitals for other purposes or healthy relatives who had never had a seizure. A neurologist assessed controls to ensure they had no history of seizures. Respondents with an affirmative response to screening questions were deemed screen-positive except if they had febrile convulsions. Cases and controls were matched for geographical location and ethnicity. We excluded those who were severely ill and on admission.

### 2.2 Study design

A cross-sectional study was conducted in the Greater Accra region between August and October 2022 at the outpatient department of selected facilities in the Shai-Osudoku and Ningo-Prampram districts and the Korle-Bu Teaching Hospital. The study is part of the Epilepsy Pathway Innovation in Africa (EPInA) project (https://epina.web.ox.ac.uk/home). The Epilepsy Pathway Innovation in Africa (EPInA) is a multi-country project funded by the UK National Institute for Health Research. It aims to improve the quality of life for people with epilepsy in sub-Saharan Africa by addressing all aspects of the treatment pathway: prevention, diagnosis, treatment, and awareness. The project began in 2019 and is scheduled to run until January 2025. The EPInA team is working in Accra, Ghana; Nairobi and Kilifi, Kenya; and Mahenge, Tanzania, to enhance these four aspects of the epilepsy pathway. In the Ghana project, the intention was to use a validated screening questionnaire to identify all forms of epilepsy in the two districts (Shai-Osudoku and Ningo-Prampram) of the Greater Region; hence, this pilot study was to test the sensitivity and specificity of the screening questions.

### 2.3 Epilepsy screening tool development

We conducted a search to identify screening tools (questionnaires) previously used in LMICs [16,20–22] to help develop our instrument. A local neurologist reviewed the initial version to arrive at the final draft. We then forward and back-translated the tool into the two targeted languages of Asante Twi and Dangme. We included questions on convulsive and non-convulsive seizures to cover as many seizure types as possible. We divided the questionnaire into two parts. Stages 1 and 2. The first part is administered to either the household head or an adult member with a good knowledge of household members, and stage 2 is administered to people positive in stage 1 (Table 1). For the validation study, the seventeen questions were administered to the cases and controls to gauge the sensitivity and specificity of the screening question.

**Table 1:** Questionnaire used for screening.

	**Stage 1** (Administered to the head of household or his/her representative)
Q1	Do you or any member of your household have fits or has someone ever told you that you/they have fits?
Q2	Do you/this member of the household experience episodes in which your/their legs or arms have jerking movements or fall to the ground and lose consciousness?
Q3	Have you/this household member experienced an unexplained change in your mental state or level of awareness; or an episode of “spacing out” that you/they could not control?
Q4^a^	Do you/any member of the household have experiences such as hallucinations or strange feelings e.g., epigastric rising and smells that are non-existent, and sudden emotional changes, such as unexplained fear, anxiety, or even déjà vu?
Q5^a^	Have you/any household member ever experienced a blank stare, unfamiliarity with surrounding and fumbling and chewing movements, and has/has no recollection of anything that happened at that time?
Q6^a^	Have you/any member of the household ever had reports of being unresponsive, or had an abrupt blank stare or interruption of ongoing activities for few seconds, sometimes with upward eye deviation/rolling
Q7	Do you/any member of the household abruptly fall on your or their head/face/buttocks/back, sometimes sustaining injuries, and waking up soon after that?
	**Stage 2** (To be administered to only those who are screened as positive from Stage 1)
Q1^b^	Did anyone ever tell you that you/this member of the household had a seizure or convulsion caused by a high fever when you were a child?
Q2	Have you/this member of the household ever been told by a doctor that you have epilepsy or epileptic fits?
Q3	Have you/this member of the household ever been told by someone else that you have epilepsy or epileptic fits? Q4. If yes, who told you?. . . . . . . . . . . . . . . . . . . . . . . . . . . . . . . . . . . . . . . . . . . . . . . . . . . . . . . . . . . . . . . . . . . . . . . . . . . . . . . .
Q5	Have you/this household member ever fallen to the ground without a reason and experienced twitching?
Q6	Have you/this household member ever fallen to the ground without a reason and wet yourself?
Q7	Have you/this household member ever fallen to the ground without a reason and bitten your tongue?
Q8^a^	Did anyone ever tell you/this household member that when you/they were a small child, you/they would daydream or stare into space more than other children?
Q9	Have you/this household member ever noticed any unusual body movements or feelings when exposed to strobe lights, flickering lights, or sun glare?
Q10	Shortly after waking up, either in the morning or after a nap, have you/this household member ever noticed uncontrollable jerking or clumsiness, such as dropping things or suddenly “flying” from your hands?
Q11	Have you/this household member ever had any other type of repeated unusual spells?

a. Non-convulsive epilepsy screening questions.

b. Childhood febrile convulsion question, excluded from the overall classification of cases as positive.

c. Q4 was an ancillary question regarding the reference person that reported the diagnosis of epilepsy of the interviewed subject.

### 2.4 Translation of the screening questionnaire to the local language

Bilingual experts from the Bureau of Ghanaian Languages were consulted to translate the questionnaire into two widely spoken languages in the study setting (Greater Accra region of Ghana): Twi and Dangme, which were back-translated by individuals fluent in the languages who were not involved in the initial translation, following the published guidance on the translation of questionnaires to ensure the meaning remained the same [23]. Jevetic Educational Services undertook the back translations. Two content experts, a local physician and a neurologist, reviewed the translated versions of the questionnaire to resolve any discrepancies before arriving at the final draft. The questionnaires were also extensively discussed at stakeholder meetings and during training of the research assistants for comprehension, socio-cultural acceptability and adaptability before they were agreed on for the final version.

### 2.5 Sensitivity and specificity of the screening questionnaire

We measured the questionnaire’s sensitivity and specificity by administering it to people with confirmed epilepsy and comparing the answers to those without epilepsy. The gold standard for a confirmed case was diagnosis by a physician/neurologist with epilepsy expertise. Four trained field workers read the questionnaires verbatim to the participants and recorded the responses. They were, however, not blinded to the diagnosis.

### 2.6 Sample size determination

The sample size was estimated using the formula for testing sensitivity (or specificity) of a single diagnostic test [24] (See S2 Appendix), which suggested that the questionnaire should be administered to at least 44 people in each language group to have 80% power with 95% confidence to measure the sensitivity and specificity.

### 2.7 Study variables

The questionnaires collected demographic data on age, sex, education, religion, ethnicity, marital status, and the screening questions. The screening questions used at the two stages of the study are shown in Table 1.

### 2.8 Data management and statistical analysis

The data collected on paper questionnaires was entered into Microsoft Excel (version 365) for quality checks and cleaning, after which it was imported into Stata statistical analysis software (version 16)[25] for analysis. Frequencies and percentages were used to describe the demographic data. The Stata module’ diag’ was used to report summary statistics for the diagnostic tests, comparing the results from the questionnaire to true disease status.

### 2.9 Classification of cases and controls from screening

For these classifications, if a respondent answered “Yes” to any of the questions used at stages 1 and 2 of the screening (see Table 1), they were deemed screen-positive for that stage, except for Question 1 from Stage 2, which is a question on febrile seizures/convulsions and was not included in such classifications. The questionnaires were divided into stages 1 and 2. Stage 1 aimed to identify potential epilepsy cases through broader questions. This stage was meant to be administered at the household level, allowing a knowledgeable household member to answer for others in their absence. We included questions on non-convulsive seizures (Q4-6), which can be challenging to self-report but may still indicate epilepsy. Stage 2 included more specific questions targeting individuals identified in Stage 1. This step was designed to reduce false positives and improve diagnostic accuracy. After these two stages, individuals who screened positive would move to Stage 3, which involved a clinical evaluation, including an EEG, to confirm the diagnosis. The study was designed to gauge the sensitivity and specificity of the screening questions, with a broader study to be conducted using the bigger Epilepsy Pathway Innovation in Africa household prevalence survey as the basis. For the validation, the 17-item screening questions were administered to cases and controls.

### 2.10 Validation of the screening questionnaire

Sensitivity was defined as the proportion of people with the condition correctly identified and specificity as the proportion of healthy individuals correctly identified. The ROC (Receiver Operating Characteristic Curve) defined the area under the curve (for a simple test) as the average of sensitivity and specificity. The positive and negative predictive values (PPV & NPV) show the individual’s probability of having the disease following a positive or a negative test. If no prevalence figure is given, the sample is assumed to be a cohort, and PPV & NPV are the proportions of test positives and test negatives identified. Otherwise, they are estimated using the likelihood ratios, assuming the prevalence is correct.

The likelihood ratio of a positive test (LR+) is the ratio of the probability (likelihood) of a positive test result in an affected and in an unaffected individual = Sensitivity/ (1- specificity). Multiplying the prior odds of disease by LR+ gives the odds of disease following a positive test. The likelihood ratio of a negative test (LR-) works similarly. The positive LR is the probability of a positive outcome having a positive screening divided by the likelihood of a negative outcome having a positive screening. The negative LR is the probability of a positive outcome with a negative screening divided by the possibility of a negative outcome having a negative screening. We assessed sensitivity and false-positive rates for the instrument by administering it to individuals with medical record–documented epilepsy or isolated unprovoked seizure and individuals who were seizure-free on medical record review from selected health facilities in two districts in the Shai-Osudoku and Ningo-Prampram districts of the Greater Accra Region of Ghana.

### 2.11 A ethical considerations

Ethical approval for this study was obtained from the Institutional Review Board of the Dodowa Health Research Centre (DHRCIRB/95/08/20) and the Ghana Health Service Ethics Review Committee (GHS-ERC 022/08/20). Informed consent was obtained from all study participants. Before their involvement in study, permission was obtained from one parent or guardian, along with assent from the participant. Participants aged 18 and over provided written informed consent.

During the consenting process, the privacy of the respondents was ensured by conducting study activities in a secluded area with minimal exposure to other individuals. The objectives, justification, and methodologies of the study, as well as the potential risks and benefits, were thoroughly explained to all participants, who were allowed to ask questions. Participation in the study was entirely voluntary. Although immediate or direct benefits were not provided to participants, it was emphasised that their contributions would aid in advancing our understanding of epilepsy and assist in developing a standardised screening tool for identifying individuals with epilepsy.

Participants were informed of their right to withdraw from the study without facing any repercussions. Responses to screening questions were solicited in a yes or no format. Subsequently, interviews were recorded in Microsoft Excel and transferred to Stata for analysis. All hard and soft data were securely stored in locked file cabinets, with access restricted to the study team. Study participants provided written informed consent.

## 3. Results

### 3.1 Background characteristics of respondents

One hundred and forty participants (70 cases and 70 controls) were recruited for the Dangme questionnaire (Table 2). Among the controls, 40% were young adolescents (age range of 10–19 years); 40% were females, and 34.3% had no formal education (Table 2). Among the cases for the Dangme version, 34.3% were aged 30–39 years; 72.9% were females, and 41.4% had completed Junior High School (JHS)/middle level of formal education (Table 2). The Asante Twi version recruited 100 people (50 cases and 50 controls). Among the 50 controls, 36% were young adults between the age range of 20–29 years, 20% were females, and 26% had completed JHS/Middle school. Among the cases for the Asante Twi version, 24% were between the ages of 30 and 39 years, 60% were females, and 22% had completed some tertiary-level education.

**Table 2:** Background characteristics of respondents.

	Dangme				Asante Twi			
	Cases [N = 70]		Controls [N = 70]		Cases [N = 50]		Controls [N = 50]	
	N	% (95% CI)	N	% (95% CI)	N	% (95% CI)	N	% (95% CI)
Age (years) <10 10–19 20–29 30–39 40–49 50–59 60+	0 2 7 24 23 6 8	- 2.9 (0.7, 11.0) 10.0 (4.8, 19.7) 34.3 (24.0, 46.3) 32.9 (22.8, 44.8) 8.6 (3.8, 18.0) 11.4 (5.7, 21.4)	6 28 13 15 5 2 1	8.6 (3.8, 18.0) 40.0 (29.1, 52.0) 18.6 (11.0, 29.6) 21.4 (13.2, 32.8) 7.1 (3.0, 16.3) 2.9 (0.7, 11.0) 1.4 (0.2, 9.8)	0 1 7 12 7 12 11	- 2.0 (0.3, 13.4) 14.0 (6.7, 27.0) 24.0 (14.0, 38.1) 14.0 (6.7, 27.0) 24.0 (14.0, 38.1) 22.0 (12.4, 35.9)	2 13 18 9 4 1 3	4.0 (1.0, 15.1) 26.0 (15.5, 40.2) 36.0 (23.7, 50.4) 18.0 (9.5, 31.5) 8.0 (3.0, 19.9) 2.0 (0.3, 13.4) 6.0 (1.9, 17.4)
Sex Female Male	51 19	72.9 (61.1, 82.1) 27.1 (17.9, 38.9)	28 42	40.0 (29.1, 52.0) 60.0 (48.0, 70.9)	30 20	60.0 (45.6, 72.8) 40.0 (27.2, 54.4)	10 40	20.0 (10.9, 33.7) 80.0 (66.3, 89.1)
Education None Primary JHS/Middle School SHS/O&A Level Tertiary	13 13 29 12 3	18.6 (11.0, 29.6) 18.6 (11.0, 29.6) 41.4 (30.4, 53.4) 17.1 (9.9, 28.0) 4.3 (1.4, 12.7)	24 21 20 4 1	34.3 (24.0, 46.3) 30.0 (20.3, 41.9) 28.6 (19.1, 40.4) 5.7 (2.1, 14.5) 1.4 (0.2, 9.8)	2 4 17 5 22	4.0 (1.0, 15.1) 8.0 (3.0, 19.9) 34.0 (22.0, 48.4) 10.0 (4.1, 22.3) 44.0 (30.7, 58.2)	4 10 13 14 9	8.0 (3.0, 19.9) 20.0 (10.9, 33.7) 26.0 (15.5, 40.2) 28.0 (17.1, 42.3) 18.0 (9.5, 31.5)
Religion Christian Other	66 4	94.3 (85.5, 97.9) 5.7 (2.1, 14.5)	67 3	95.7 (87.3, 98.6) 4.3 (1.4, 12.7)	47 3	94.0 (82.6, 98.1) 6.0 (1.9, 17.4)	47 3	94.0 (82.6, 98.1) 6.0 (1.9, 17.4)
Ethnicity Akan Ewe Ga-Dangbe Other	5 2 58 5	7.1 (3.0, 16.3) 2.9 (0.7, 11.0) 82.9 (72.0, 90.1) 7.1 (3.0, 16.3)	5 2 60 3	7.1 (3.0, 16.3) 2.9 (0.7, 11.0) 85.7 (75.2, 92.2) 4.3 (1.4, 12.7)	22 9 17 2	44.0 (30.7, 58.2) 18.0 (9.5, 31.5) 34.0 (22.0, 48.4) 4.0 (1.0, 15.1)	21 8 18 3	42.0 (28.9, 56.3) 16.0 (8.1, 29.3) 36.0 (23.7, 50.4) 6.0 (1.9, 17.4)
Marital status Single/Underage Married Divorced/Separated/ Widowed	14 47 9	20.0 (12.1, 31.2) 67.1 (55.2, 77.2) 12.9 (6.7, 23.1)	59 7 4	84.3 (73.6, 91.2) 10.0 (4.8, 19.7) 5.7 (2.1, 14.5)	10 30 10	20.0 (10.9, 33.7) 60.0 (45.6, 72.8) 20.0 (10.9, 33.7)	40 8 2	80.0 (66.3, 89.1) 16.0 (8.1, 29.3) 4.0 (1.0, 15.1)

Table 3 provides the classification of cases and controls from screening. Seventy (100%) with confirmed epilepsy screened positive on all items at stage 1, and only one confirmed epilepsy case screened negative on all item questions at stage 2. In comparison, 14 (20%) and 10 (14.28%) controls screened positive at stage 1 and 2 in the Dangme questionnaire. One (2%) with confirmed epilepsy screened negative on all items at stage 1, and 2 (4%) with confirmed epilepsy screened negative on all item questions at stage 2. In comparison, among the controls, 4 (8%) and 3 (6%) screened positive on all item questions at stages 1 and 2 in the Asante Twi questionnaire.

**Table 3:** Classification of cases and controls from screening.

	Dangme (questionnaire)			Asante Twi (questionnaire)		
	+ve	-ve	Total	+ve	-ve	Total
Stage 1						
True +ve	70	0	70	49	1	50
True -ve	14	56	70	4	46	50
Total	84	56	140	53	47	100
Stage 2						
True +ve	69	1	70	48	2	50
True -ve	10	60	70	3	47	50
Total	79	61	140	51	49	100

*** +ve: Positive and -ve: Negative.

Sensitivities, specificities, PPVs and NPVs of the overall questionnaire are shown in Table 4. The overall predictive ability of both questionnaires was good. The overall performance was 100% sensitivity and specificity 80% for the Dangme questionnaire at stage 1 and 98.6% and 85.7% at stage 2. The overall PPV and NPV at stage 1 was 83.3% and 100%, and 87.3% and 98.4% at stage 2. The Asante Twi version’s overall sensitivity was 98%, and specificity was 92% at stage 1. At stage 2, the sensitivity was 96% and specificity was 94%. The PPV and NPV were above 90%, and the same for stage 2 of the questionnaire.

**Table 4:** Validation of the overall classifications from the questionnaire compared to true disease status.

	Dangme			Asante Twi		
	Test	95% CI		Test	95% CI	
	value	Lower	Upper	value	Lower	Upper
Stage 1						
Prevalence (%)	50.0	41.0	58.6	50.0	40.0	60.2
Sensitivity (%)	100.0	88.6	94.9	98.0	89.4	99.9
Specificity (%)	80.0	68.7	88.6	92.0	80.8	97.8
Positive predictive value (%)	83.3	73.6	90.6	92.5	81.8	97.9
Negative predictive value (%)	100.0	93.6	100.0	97.9	88.7	99.9
ROC area	0.90	0.85	0.95	0.95	0.91	0.99
Likelihood ratio (+)	5.00	3.13	7.99	12.30	4.78	31.40
Likelihood ratio (-)	0.00	-	-	0.02	0.00	0.15
Stage 2						
Prevalence (%)	50.0	41.0	58.6	50.0	40.0	60.2
Sensitivity (%)	98.6	92.3	100.0	96.0	86.3	99.5
Specificity (%)	85.7	75.3	92.9	94.0	83.5	98.7
Positive predictive value (%)	87.3	78.0	93.8	94.1	83.8	98.8
Negative predictive value (%)	98.4	91.2	100.0	95.9	86.0	99.5
ROC area	0.92	0.88	0.97	0.95	0.91	0.99
Likelihood ratio (+)	6.90	3.88	12.30	16.00	5.33	48.00
Likelihood ratio (-)	0.02	0.00	0.12	0.04	0.01	0.17

Individual questions’ sensitivity, specificity, PPVs, and NPVs are shown in Table 5. For the Dangme version at stage 1, the questions Q1-Q7 had a good sensitivity, while Q1, Q2, Q5, and Q7 had the best sensitivity overall. The sensitivity ranged between 91.5% and 97.1%. The specificity was between 91.4% and 95.7% for all individual questions. With the questions at stage 2, the sensitivity was lower with some individual questions compared to the stage 1 individual questions, with most questions having a sensitivity of 60% and below, apart from Q2 (94.3%), Q3 (85.7%), and Q5 (94.3%). In the Asante Twi version, all the questions had good sensitivity except Q5, which was 38% at stage 1. The specificity was between 96% and 100% for all individual questions. With the questions at stage 2, the sensitivity was lower in the Twi version, with most questions having a sensitivity of 54% and below, apart from questions Q2 and Q5, with 84% and 82%. The specificity was between 96% and 100%.

**Table 5:** Validation of the individual screening questions compared to true disease status.

	Dangme, %				Asante Twi, %			
Question	Se	Sp	PPV	NPV	Se	Sp	PPV	NPV
Stage 1								
Q1	97.1	95.7	95.8	97.1	82.0	100.0	100.0	84.7
Q2	94.3	91.4	91.7	94.1	82.0	100.0	100.0	84.7
Q3	84.3	95.7	95.2	85.9	76.0	96.0	95.0	80.0
Q4^a^	81.4	91.4	90.5	83.1	64.0	100.0	100.0	73.5
Q5^a^	91.4	91.4	91.4	91.4	38.0	100.0	100.0	61.7
Q6^a^	87.1	91.4	91.0	87.7	68.0	96.0	94.4	75.0
Q7	92.9	94.3	94.2	93.0	68.0	100.0	100.0	75.8
Stage 2								
Q1^b^	47.1	87.1	78.6	62.2	32.0	100.0	100.0	59.5
Q2	94.3	97.1	97.1	94.4	84.0	100.0	100.0	86.2
Q3	85.7	94.3	93.8	86.8	66.0	100.0	100.0	74.6
Q5	94.3	100.0	100.0	94.6	82.0	96.0	95.3	84.2
Q6	61.4	100.0	100.0	72.2	54.0	100.0	100.0	68.5
Q7	58.6	100.0	100.0	70.7	60.0	100.0	100.0	71.4
Q8	60.0	97.1	95.5	70.8	18.0	100.0	100.0	54.9
Q9	35.7	98.6	96.2	60.5	26.0	100.0	100.0	57.5
Q10	41.4	97.1	93.5	62.4	40.0	100.0	100.0	62.5
Q11	41.4	95.7	90.6	62.0	22.0	98.0	91.7	55.7

= Sensitivity, Sp = Specificity, PPV = Positive predictive value, NPV = Negative predictive value.

Non-convulsive epilepsy screening questions.

Childhood febrile convulsion question, excluded from overall classification of cases as positive.

## 4. Discussion

We translated and validated an epilepsy screening questionnaire into two Ghanaian languages, which showed good sensitivity, specificity, PPV, and NPV. We explored the differences in individuals’ sensitivities and specificities and the overall component items of the questionnaire. The likelihood ratios indicated a reasonable but varied probability of predicting epilepsy. These screening tools are simple and can be easily used by health workers at the community level, suggesting a robust epilepsy screening tool for Ghana in these languages. There was limited difference in the specificity of individual and overall item questions, but we noted significant differences in the sensitivity across some individual questions (Tables 4 and 5). Specificity measures were also consistent between questions and languages. This means that a positive screen was consistent with a diagnosis of epilepsy”.

We conducted a hospital-based validation of confirmed epilepsy cases compared to controls. It is known that the diagnostic accuracy of such a study design is usually inflated. Cases with more severe forms of the condition and greater awareness of their state of health have a higher chance of being included. These people will likely screen positive with this instrument, possibly leading to overestimating the sensitivity [26]. Consequently, there is a risk of obtaining inaccurate estimates of the disease prevalence when applying a hospital-based validated questionnaire to the general population. The advantage of validation, however, is that the prevalence can be adjusted to the sensitivity and specificity of the screening test [27].

We also noticed some variations in the accuracy measures between the two languages. This might be due to differences in population characteristics, methods of administration of the questionnaires between the various facilities, linguistic features, and translation dynamics [22,28]. The differences could be attributed to variations in the general literacy and epilepsy awareness and stigma between the two populations. Health facility-based validation studies are less influenced by stigma-related concealment than community- or population-based studies [29]. The differences in sensitivity of some of the individual item questions may also relate to the frequency of the phenomena (e.g., incontinence, absence seizures, jerking movements or falling to the ground). Absence seizures or non-convulsive seizures may often be overlooked by people living with the condition. Low sensitivity of non-convulsive seizure detection has been previously reported [30].

Currently, no comparable studies are available in Ghana using similar screening questionnaires. A study in Nigeria using a nine-item questionnaire reported similar sensitivity and specificity with a slightly lower PPV [22]. A study in Ecuador had a low PPV of 18.3% [20]. The Rochester Epidemiology project also had a PPV of 23% [21]. Our study had a relatively higher PPV, indicating that more people with epilepsy were likely to screen positive.

An advantage of this screening tool is that it includes questions that address multiple seizure types, unlike other available screening tools that address only convulsive epilepsy. A further benefit of our study is the estimation of likelihood ratios for the two languages. This is crucial because sensitivity and specificity alone may not provide enough information on the probability of diagnosing epilepsy. Likelihood ratios are more appropriate for comparing individual questions [31].

We found no difficulties in training a local person to administer the screening questionnaires in the two local languages or any challenges with comprehension of the questions. This was mainly because the data collectors were natives who understood the test languages and administered them to respondents who spoke and understood the same.

Finally, blinding is crucial in diagnostic validation studies to minimize bias and ensure the accuracy of reported sensitivity and specificity estimates. In epilepsy validation studies, blinding helps prevent data collectors’ knowledge of participants’ prior diagnoses from influencing how screening tools are administered or interpreted. In our study, data collectors were not blinded, which could have introduced bias, potentially affecting the accuracy of sensitivity and specificity by influencing question delivery or symptom interpretation. Studies suggest that lack of blinding can lead to diagnostic expectation bias and observer bias, inflating diagnostic accuracy, especially for subjective conditions like epilepsy [32,33]. To address this in future studies, strict blinding protocols should be used, ensuring data collectors are unaware of participants’ medical histories. Standardized tools, such as pre-recorded audio or video questionnaires, could further reduce interviewer bias. Although this study did not implement blinding, efforts were made to minimize bias by training data collectors to administer questions verbatim. However, future studies should follow the Standards for Reporting Diagnostic Accuracy (STARD) guidelines [34] and include blinding to enhance study rigor and reliability.

## 5. Limitations

This study was conducted within healthcare facilities. This could result in selection bias as questions were administered to people with confirmed epilepsy who could differ from the general population. This selection bias limits the overall generalizability of the results. Our approach could lead to an underestimation of epilepsy at the community level, as less overt cases may be missed. The lack of awareness of epilepsy may result in people failing to seek treatment; these people may not have been captured in our study. Our eventual goal is to validate the tool for use in the general population. Here, information was collected at the facility level and from the cases about their medical histories rather than their relatives. Some individuals might be unaware of specific symptoms, which could explain why some cases screened negative. The use of proxies or caregiver questions was an option we considered for stage 1 of our screening tool. We did not explore this further as some cases did not involve caregivers, and we wanted to ensure consistency across data capture. Future research on the use of proxies or relatives to respond to on behalf of cases would be helpful to see if this can improve reliability, particularly for people with non-convulsive seizures.

## 6. Conclusion

We have validated a screening tool for epilepsy that trained community health workers can use to screen for epilepsy in communities in Ghana. Our tool could be adapted to other sub-Saharan African countries following appropriate validation.

## Supporting information

S1 Appendix Demographic questionnaire. (PDF)

S2 Appendix Formula for calculating sample siz. (DOCX)

S3 Appendix EPInA screening validation dataset. (CSV)

S4 Appendix Screening questionnaire. (PDF)
