# Supplementary material for: Translation, adaptation and validation of an epilepsy screening instrument in two Ghanaian languages
Source: PLoS One. 2025 Jan 17;20(1):e0303735. doi: 10.1371/journal.pone.0303735 (PMC11741578; doi:10.1371/journal.pone.0303735)
Supplement: S1 Appendix — (PDF) [file pone.0303735.s001.pdf]

DODOWA HEALTH RESEARCH CENTRE

EPILEPSY PATHWAY INNOVATION IN AFRICA

VALIDATION STUDY

ALL QUESTIONS AT EACH STAGE MUST BE ASKED TO THE ELIGIBLE RESPONDENTS

FIELDWORKER CODE

RESPONDENT CODE

HEALTH FACILITY CODE

INTERVIEW DATE

DEMOGRAPHIC DATA ON STUDY PARTICIPANTS

1. NAME …….
2. AGE IN YEARS…….
3. DATE OF BIRTH

4. PLACE OF BIRTH………………………………………………………………………………………………………
5. PLACE OF RESIDENCE……………………………………………………………………………………………….
6. LENGTH OF STAY………………………………………………………………………………………………………
7. HIGHEST EDUCATIONAL LEVEL

1. None	2. Primary	3. Junior High Sch.	4. Senior High School / Vocation	5.Tertiary	6. Others (Specify)

8. RELIGION

1. Traditional	2. Islamic	3. Christian	4 others(specify)

9. ETHNICITY

1. Akan	2. Ewe	3. Ga-Adangme	4. Northern	5.Others (specify)

10. OCCUPATION

1.Unemployed	2. Student	3.Farmer	4.Fisherman	5.Artisan	6.Civil Servant	7. Others (Specify

11. MARITAL STATUS

1.Married	2.Widowed	3.Single	4.Separated	5.Divorced	6. Others (specify)
