# Supplementary material for: Translation, adaptation and validation of an epilepsy screening instrument in two Ghanaian languages
Source: PLoS One. 2025 Jan 17;20(1):e0303735. doi: 10.1371/journal.pone.0303735 (PMC11741578; doi:10.1371/journal.pone.0303735)
Supplement: S2 Appendix — (DOCX) [file pone.0303735.s002.docx]

**S2 Appendix: The formula for sample size Calculation**

Where $P_{0}$ is the pre-determined value of sensitivity (or specificity) of the diagnostic questionnaire in English set at 98%, $P_{1}$ is the expected sensitivity (or specificity) of each of the Asante Twi and Dangme translated questionnaires also set at 90%, $Z_{\frac{\alpha}{2}}$ = 1.96 and $Z_{\beta}$ = 0.84 are critical values from the standard normal distribution for a 95% confidence level and 80% power in estimating differences in diagnostic capabilities of the tools compared to the English one. Using the above figures, the minimum sample size required for each group (case or control) for the Asante Twi and Dangme languages was 44 persons.
