# Supplementary material for: Translation, adaptation and validation of an epilepsy screening instrument in two Ghanaian languages
Source: PLoS One. 2025 Jan 17;20(1):e0303735. doi: 10.1371/journal.pone.0303735 (PMC11741578; doi:10.1371/journal.pone.0303735)
Supplement: S4 Appendix — (PDF) [file pone.0303735.s004.pdf]

DODOWA HEALTH RESEARCH CENTRE

EPILEPSY PATHWAY INNOVATION IN AFRICA

EPILEPSY SCREENING QUESTIONNAIRE

VALIDATION STUDY

ALL QUESTIONS AT EACH STAGE MUST BE ASKED TO THE ELIGIBLE RESPONDENTS

FIELDWORKER CODE

RESPONDENT CODE

HEALTH FACILITY CODE

INTERVIEW DATE

	**Stage 1** (To be administered to the CASES AND CONTROLS)
Q1	Do you/this member of the household have fits or has someone ever told you that you/they have fits? YES/NO
Q2	Do you/this member of the household experience episodes in which your/their legs or arms have jerking movements or fall to the ground and lose consciousness? YES/NO
Q3	Have you/this member of the household experienced an unexplained change in your mental state or level of awareness; or an episode of "spacing out" that you/they could not control? YES/NO
Q4*	Do you/any member of the household have experiences such as hallucinations or strange feelings e.g., epigastric rising and smells that are non-existent, and sudden emotional changes, such as unexplained fear, anxiety, or even déjà vu? YES/NO
Q5*	Have you/any member of the household ever experienced a blank stare, unfamiliarity with surrounding and fumbling and chewing movements, and have/has no recollection of anything that happened at that time? YES/NO
Q6*	Have you/any member of the household ever had reports of being unresponsive, or had an abrupt blank stare or interruption of ongoing activities for few seconds, sometimes with upward eye deviation/rolling YES/NO
Q7*	Do you/any member of the household abruptly fall on your or their head/face/buttocks/back, sometimes sustaining injuries, and waking up soon thereafter? YES/NO

Stage 2 (To be administered to CASES AND CONTROLS)

Q1 Did anyone ever tell you that you/this member of the household had a seizure or convulsion caused by a high fever when you were a child? YES/NO

Q2 Have you/this member of the household ever been told by a doctor that you have epilepsy or epileptic fits? YES/NO

Q3 Have you/this member of the household ever been told by someone else that you have epilepsy or epileptic fits? YES/NO

4. If yes, who told you? ………………………………

Q5 Have you/this member of the household ever fallen to the ground without a reason and experienced twitching?

Q6 Have you/this member of the household ever fallen to the ground without a reason and wet yourself? YES/NO

Q7 Have you/this member of the household ever fallen to the ground without a reason and bitten your tongue? YES/NO

Q8 Did anyone ever tell you/this member of the household that when you/they were a small child, you/they would daydream or stare into space more than other children? YES/NO

Q9 Have you/this member of the household ever noticed any unusual body movements or feelings when exposed to strobe lights, flickering lights, or sun glare? YES/NO

Q10 Shortly after waking up, either in the morning or after a nap, have you/this member of the household ever noticed uncontrollable jerking or clumsiness, such as dropping things or things suddenly "flying" from your hands? YES/NO

Q11 Have you/this member of the household ever had any other type of repeated unusual spells?

* Non-convulsive epilepsy screening questions? YES/NO

**A "yes" response to any of the questions will be considered as a positive screen*
